# The impact of aging and reaching movements on grip stability control during manual precision tasks

**DOI:** 10.1186/s12877-021-02663-3

**Published:** 2021-12-15

**Authors:** Bor-Shing Lin, Shu-Fen Kuo, I-Jung Lee, Liang-Hsuan Lu, Po-Yin Chen, Pin-Chun Wang, Chien-Hung Lai, Xin-Miao Wang, Chueh-Ho Lin

**Affiliations:** 1 Department of Computer Science and Information Engineering, College of Electrical Engineering and Computer Science, National Taipei University, New Taipei City, Taiwan, ROC; 2 School of Nursing, College of Nursing, Taipei Medical University, Taipei, Taiwan, ROC; 3 Department of Physical Therapy and Assistive Technology, National Yang-Ming University, Taipei, Taiwan, ROC; 4 Vitality and Ageing, Leiden University Medical Center, Leiden, Netherlands; 5 Erasmus University Rotterdam, Rotterdam, Netherlands; 6 Department of Physical Medicine and Rehabilitation, School of Medicine, College of Medicine, Taipei Medical University, Taipei, Taiwan; 7 Faculty of Humanities, Zhejiang Dong Fang Polytechnic Collage, Wenzhou, China; 8 Master Program in Long-Term Care, College of Nursing, Taipei Medical University, 250 Wu-Xing Street, 11031 Taipei, Taiwan, ROC; 9 Center for Nursing and Healthcare Research in Clinical Practice Application, Wan Fang Hospital, Taipei Medical University, 250 Wu-Xing Street, 11031 Taipei, Taiwan, ROC

**Keywords:** Older adults, Grip-stability control, Forward-reach, Hand strength, Aging, Dual task

## Abstract

**Background:**

Operating an object by generating stable hand-grip force during static or dynamic posture control of the upper extremities simultaneously is an important daily activity. Older adults require different attentional resources during grip strength control and arm movements. However, the impact of aging and reaching movements on precise grip strength and stability control among older adults is not well understood. This study investigated the impact of aging and reaching movements on grip strength and stability control in both hands of the upper extremities.

**Methods:**

Fifty healthy young adults (age: 28.8 ± 14.0 years) and 54 healthy older adults (73.6 ± 6.3 years) were recruited to perform isometric grip strength test at 20% maximal voluntary contraction as the target force during three manual precision tasks simultaneously: stationary task (without arm movements), forward-reach task, and backward-reach task. The average grip force (in kg) and coefficient of variation values (expressed as a percentage) during manual precision tasks were calculated to determine the quality of participants’ grip strength. The deviation error, absolute error, and force-stability index values were calculated to determine the strength control relative to the target force.

**Results:**

For both the young and older groups, the force-stability index values in both hands were significantly higher during forward- and backward-reaching movements than in the stationary condition (*p* < 0.05). The older group exhibited a significantly lower hand-grip strength and stability of strength control in both hands than the young group (*p* < 0.05).

**Conclusions:**

Aging and reaching task performance reduced the grip strength of participants and increased the variations in strength control of both hands relative to the target force, indicating that older adults exhibit poor grip strength and stability control when performing arm-reaching movements. These findings may help clinical therapists in establishing objective indexes for poor grip-stability control screening and developing appropriate rehabilitation programs or health-promotion exercises that can improve grip strength and stability control in older people.

## Background

Operating an object by generating stable hand-grip force during static or dynamic posture control of the upper extremities simultaneously is an important daily activity requiring different attentional resources during grip-strength control and arm movements. For example, when drinking water from a bottle, the initial focus may be on exerting sufficient grip strength to maintain the grip on the bottle and prevent it from slipping from grasp. When the grip is steady, the attention would then shift to the arm movement that may result in decreased grip-strength control. During this manual precision task, applying a constant and stable grip strength using specific force and in relation to an object’s weight requires the cooperation of perception feedback, grip strength output, arm coordination control, and cognitive function (attention and working memory) [1]. Among healthy young people, studies revealed that grip strength and moment are affected and show variable grip-force control when positioning in different postures or performing arm movements [2–5]. Furthermore, grip strength of participants in relation to an object’s load is affected at different static arm postures or during dynamic movements [2–5]. Concurrent activity combining cognition and movement control has been reported as a type of postural and suprapostural task and is an example of the dual tasks performed in previous studies [6–8]. However, describing the mechanisms involved during actual grip-strength control while performing forward and backward-reaching movements in daily activities is difficult because the different tasks, involving arm postures and movements, may require various central processing resources and could lead to a different impact on grip-strength control. There are scarce studies of this type focusing on older people.

For older adults, many studies have found that the degeneration of the neuromuscular physiology associated with aging not only results in decreasing muscle mass, strength, and perception feedback [9–11] but also further affects functional performance [12, 13]. Therefore, many studies have evaluated the maximal grip-strength and used it as an indicator for screening and predicting frailty, illness, sarcopenia, and associated disability and mortality in older adults [14–17]. Based of clinical observation, we also found that many healthy older adults show normal grip-strength performance when a dynamometer is used to evaluate their instant maximal voluntary contraction (MVC); however, these subjects cannot grasp and hold objects stably when performing normal daily activities. Additionally, most daily-life activities involving the upper extremities are a combination of steady and continuous submaximal grip strength output and arm movements, rather than the sudden generation of maximal grip strength. Therefore, the investigation in older adults of age-related changes in the course of continuous, precise, and stable submaximal grip-strength control and in relation to an object’s weight during arm-reaching movements may help to elucidate the effects of aging and arm movement on grip-strength control during activities of daily living. Thus, this study aimed to investigate the impact of aging and forward- and backward-reaching movements on grip-stability control by performing manual precision tasks. We hypothesized that grip-strength control of older adults in relation to the target force is affected by arm-reaching movements and aging. The findings of this study may be valuable to clinical therapists in developing appropriate health-promotion exercises or rehabilitation programs to improve grip-strength control during arm movements and prevent aging-related disabilities in the future.

## Methods

### Study design and experimental procedures

This was a cross-sectional study. The independent variables considered in the analysis were hand dominance, age, and manual precision tasks. Before commencing the study, the participants were asked to sit on a high, fixed chair in front of a table, use their dominant or non-dominant hand to hold the digital electronic hand-held dynamometer in the starting position, and watch the LCD screen on the table. Data collection for hand-grip strength during the three manual precision tasks (arm stationary and forward- and backward-reaching movements) was based on a modified version of the clinical evaluation protocol of the American Society of Hand Therapists [18]. In the starting position, the shoulder joints of the tested upper limb were at 40°–50° adduction in the horizontal plane and at 30°–40° flexion in the sagittal plane, with the elbow at 90°–110° flexion, the forearm in the neutral position, and the wrist at 0–30° of dorsiflexion [19]. Before grip-force stability control was assessed during the three manual precision tasks, the MVC test was conducted by asking all the participants to grasp the dynamometer with their maximum grip force; this task was performed for both hands. The MVC values for both hands were confirmed. The data collection and study procedure for the MVC test followed a protocol described in previous studies [20]. Abnormal compensatory movement and fatigue were avoided during grip-force generation by using the lower of the two (dominant and non-dominant hand) MVC values and calculating the 20% MVC as the target grip force of each participant. The 20% MVC value was used as the target grip force because previous studies have reported that higher target grip force (30% MVC) could induce bradycardia, overloading of muscle activities, and fatigue [21], whereas a lower target grip force (5% or 10% MVC) may cause unstable grip-force stability control in both young and older adults [12, 22]. After participants completed the grip-force stability control tests during the three manual precision tasks, the data were analyzed by a statistician who was blinded to the conditions and groups.

### Subjects

The total sample size of 104 participants for this research was calculated using G*Power (Version 3.1.9.2), at 80% power, a medium effect size of 0.5, and a confidence interval of 95% (α = 5%). Based on this calculation, 50 healthy young adults (age: 28.8 ± 14.0 years; 33 female and 17 male; 44 right-handed) and 54 healthy older adults (age: 73.6 ± 6.3 years; 46 female and 9 male; 53 right-handed) from colleges and communities who fulfilled the inclusion criteria were selected between 2017 and 2018 by using convenience sampling. The inclusion criteria for both young and older participants were as follows: (1) good health and normal cognition, (2) absence of diseases that could affect hand-grip strength control or functional forward- and backward-reaching movements for bilateral upper extremities, and (3) ability to understand the instructions and the procedures. The mental status in the older group was determined using the Mini-Mental Status Examination (MMSE) to exclude the effect of age-related decline in mental status on grip-force stability control scores during reaching movements [23]; an MMSE score of 24 or higher was required for inclusion in the older adult group [24]. The exclusion criteria were as follows: (1) presence of cognitive deficits, (2) presence of a degenerative neurologic disease that could affect grip strength and functional performance of the upper limbs, (3) presence of an acute or chronic neuromuscular disorder affecting both the hands and the upper extremities, and (4) pain or discomfort while generating grip force during the reaching movements. This study was approved by the Institutional Review Board of Taipei Medical University (approval no. N201704083), and all subject recruitment and data collection describes in the methods were performed in accordance with the study protocol. Informed consent forms were signed by each participant. Furthermore, each participant’s dominant hand was identified by observing the hand used to sign the informed consent form.

### Research device and data processing

Changes in hand-grip control during three manual precision tasks were recorded continuously using a digital electronic hand-held dynamometer (Hoggan Health Industries, West Jordan, UT) to evaluate muscle-strength performance. This measurement tool has excellent reliability for muscle-strength measurement [25] and has been validated in terms of its ability to generate output grip data and analyze the impact of aging on hand-grip control among older people [10, 26]. This portable electronic hand-held dynamometer can evaluate maximal grip strength up to 68 kg and perform continuous data collection during a test, which is helpful for therapists and researchers to analyze grip strength data and monitor changes during grip-force generation. In the present study, grip-force data collected using the hand-held dynamometer were transmitted via Bluetooth to a USB key (receiver) and shown real-time on a laptop via the TBS program (version 11.0.1, Hoggan Health Industries 2001). The real-time grip-force value was shown to the participants. The sampling frequency was set at 100 Hz.

### Grip-force stability control tests for the three manual precision tasks

**Fig. 1:**
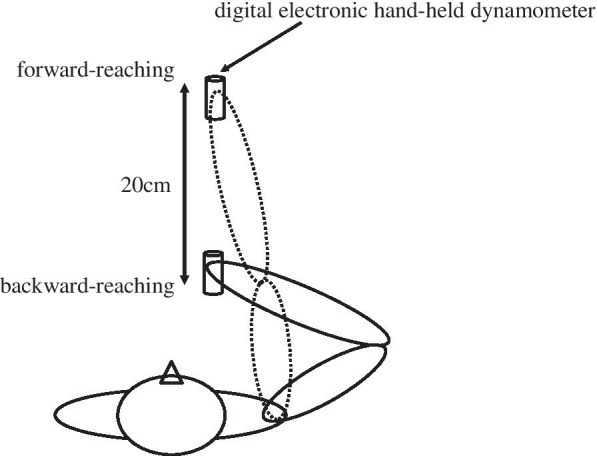
Experimental setting

Three manual precision tasks were performed for the grip-force stability control test: the stationary condition, the forward-reach condition, and the backward-reach condition (Fig. 1). Each condition involved three procedures. Each participant was asked to hold the dynamometer lightly (with either the dominant or non-dominant hand, depending on the randomization). The real-time grip data were shown to the participants through an LCD screen. The procedure for the stationary condition test was as follows: first, when a verbal “start” cue was given by the researcher, the participants began to increase their grip force until they achieved the target force. Second, another verbal cue, “hold,” was given, prompting the participants to stabilize their grip force at the target force as much as possible without any arm movement for 30 s. Finally, at the “stop” cue, the participants released their grip force on the dynamometer. The procedures for the forward-reach condition were similar to those for the stationary condition, except that arm movements were included in the second procedure. Specifically, at the verbal cue “move,” the participants moved their tested arm from the starting position to the full-reach position (shoulder at 90° adduction in the horizontal plane and 90° flexion in the sagittal plane, with the elbow at full extension) over a period of 30 s. For the backward-reach condition, all procedures were similar to the stationary condition, but the first procedure was performed at a full-reach position. Then, in the second procedure, the “back” cue prompted the participants to move their tested arms from the full-reach position back to the starting position slowly in a period of 30 s. A recent study has indicated that visual feedback can significantly improve grip strength and strength-stability control in older adults by compensatory reactions via visual information input [10]. Therefore, during the second procedure, during all three manual precision tasks, the LCD screen was covered to avoid the potential effect of visual feedback on grip strength and strength-stability control. The participants maintained and stabilized the grip force generated at the target force without knowing their force values. All grip-force data generated during the second procedure were collected and analyzed for each condition. Before data collection, each participant had one opportunity to practice the grip-force stability control required during the three manual precision tasks.

### Outcome measurements for manual precision tasks

Several parameters of grip-strength performance as the dependent variables were calculated and analyzed to reflect the impact of age and manual precision tasks on grip-force stability control in both young and older adults, including: the grip force and coefficient of variation (CV) for grip-force generation, the deviation error and absolute error (in kg), and the force-stability index for strength-stability control. The average grip force values in kg and CV (SD/mean*100%) in percentages for grip-force generation were calculated during the second procedure for each task, which represented participant grip-force stability control [10]. Additionally, the tendency and accuracy of participant grip-force stability control relative to the target force were determined by calculating the deviation error (deviation error = grip force value from subject — target force value) and absolute error (|grip force value from subject — target force value|) values in kg. The force-stability index [(variation error value/target force value ) ∗ 100%] values, expressed as percentages, were also calculated and represented the variability in grip-strength stability control relative to the target force [10]. A lower force-stability index indicates better grip-stability control at the target force level [10].

### Statistical analysis

An independent sample t-test and one-way ANOVA were used to compare the differences in participant grip strength performance (grip force and CV values) and stability control at the target force (deviation error, absolute error, and force-stability index values) for independent variables, including group, hand dominance, and manual precision task, in all samples. The Scheffe post-hoc test was applied to indicate the specific changes in grip strength performance and strength-stability control during the different manual precision task according to group, hand dominance, and manual precision task. Then, a two-way mixed ANOVA analysis was performed to examine the main and interaction effects of reaching movement and aging on grip strength performance and strength-stability control. An independent sample t-test was conducted to compare age-related changes in grip strength performance and stability control in both hands between young and older adult groups. A one-way repeated ANOVA was conducted to compare respective changes in grip strength performance and strength-stability control in each dominant and non-dominant hand under the different conditions. The Scheffe post-hoc test was applied to compare the specific changes in grip strength performance and strength-stability control for both dominant and non-dominant hands under the different conditions for each group. Based on this statistical information, we can demonstrate the effect of aging and arm-reaching movements on hand-strength stability control. The F-test sphericity assumption was validated, and the alpha level was set at 0.05. The statistical software used for the analyses was SPSS version 19.0 (SPSS Inc., Chicago, IL, USA).

## Results

Mixed model ANOVA showed a significant interaction between group and manual precision tasks for grip force (F_(5,618)_ = 18.016, *p* < 0.001, η^2^ = 0.120), CV (F_(5,618)_ = 31.817, *p* < 0.001, η^2^ = 0.198) values of muscle-strength performance, deviation error (F_(5,618)_ = 18.525, *p* < 0.001, η^2^ = 0.123), absolute error (F_(5,618)_ = 6.100, *p* < 0.001, η^2^ = 0.390), and force-stability index (F_(5,618)_ = 49.759, *p* < 0.001, η^2^ = 0.281) values of strength-stability control. Further analysis of the interaction effects of reaching movement and aging on grip stability control revealed that the older adults exhibited the highest CV (*p* < 0.001) and force-stability index (*p* < 0.001) under the backward-reach condition.

### Effect of group, hand dominance, and manual precision task on muscle-strength performance and stability control

Results showed that grip force for the muscle-strength performance and deviation error for strength-stability control in the older adult group were significantly decreased relative to those in the young group (all *p* < 0.001; Table 1). The CV and the force-stability index values in the older adult group were significantly greater than those observed in the young adult group (all *p* < 0.001; Table 1). In addition, we also found that the different reaching movements significantly affected all indices for strength performance and strength-stability control (grip force F_(2,621)_ = 3.893, *p* = 0.021; CV F_(2,621)_ = 14.152, *p* < 0.001; deviation error F_(2,621)_ = 3.967, *p =* 0.019; absolute error F_(2,621)_ = 10.380, *p* < 0.001; force-stability index F_(2,621)_ = 52.498, *p* < 0.001, respectively). Post-hoc analysis revealed that grip force and deviation error values under the forward-reach conditions were significantly higher than those under the backward-reach condition (*p* = 0.025, *p* = 0.028), and the CV, absolute error, and force-stability index values under the forward- and backward-reach conditions were significantly higher than those under the stationary condition (all *p* < 0.001). However, there was no significant difference in terms of grip-strength performance and stability control for hand dominance between both hands.

**Table 1:** The effect outcomes of group, hand dominance, and manual precision tasks on strength performance and strength-stability control

Variables	Strength performance				Strength-stability control					
	Grip force (kg)		CV (%)		Deviation error (kg)		Absolute error (kg)		Force-stability index (%)	
	M ± SD	t (*p*)/F(*p*)/Scheffe post-hoc	M ± SD	t (*p*)/F(*p*)/Scheffe post-hoc	M ± SD	t (*p*)/F(*p*)/Scheffe post-hoc	M ± SD	t (*p*)/F(*p*)/Scheffe post-hoc	M ± SD	t (*p*)/F(*p*)/Scheffe post-hoc
**Group**		8.746 (< 0.001)**		−10.263 (< 0.001)**		8.824 (< 0.001)**		0.258 (0.796)		−10.338 (< 0.001)**
Young	2.24 ± 0.42		7.86 ± 4.73		0.25 ± 0.43		0.36 ± 0.34		10.52 ± 7.92	
Older adult	1.93 ± 0.46		13.99 ± 9.56		−0.06 ± 0.46		0.35 ± 0.30		19.85 ± 14.00	
**Hand dominance**		0.517 (0.605)		−0.614 (0.540)		0.678 (0.498)		−0.702 (0.483)		−0.560 (0.575)
Non-Dominant	2.09 ± 0.46		10.84 ± 7.53		0.09 ± 0.47		0.35 ± 0.33		15.09 ± 12.79	
Dominant	2.07 ± 0.47		11.25 ± 8.85		0.07 ± 0.47		0.37 ± 0.31		16.65 ± 11.99	
**Manual precision task**		3.893 (*p* = 0.021)*		14.152 (*p* < 0.001)**		3.967 (0.019)*		10.380 (< 0.001)**		52.498 (< 0.001)**
1. Stationary condition	2.09 ± 0.38	3 < 2 (*p* = 0.025)*	8.64 ± 11.02	1 < 2 (*p* < 0.001)** 1 < 3 (*p* < 0.001)**	0.10 ± 0.40	3 < 2 (*p* = 0.028)*	0.28 ± 0.31	1 < 2 (*p* = 0.005)** 1 < 3 (*p* < 0.001)**	8.82 ± 9.88	1 < 2 (*p* < 0.001)** 1 < 3 (*p* < 0.001)**
2. Forward-reach condition	2.13 ± 0.48		12.02 ± 5.29		0.13 ± 0.48		0.38 ± 0.32		17.59 ± 12.33	
3. Backward-reach condition	2.01 ± 0.52		12.49 ± 6.69		0.01 ± 0.52		0.41 ± 0.31		19.69 ± 12.05	

Note. *CV* coefficient of variation, *M ± SD* mean ± standard deviation, t(*p*)/F(*p*) t-test (*p* value)/one-way ANOVA (*p* value)

*Significant difference *p* < 0.05

**Significant difference *p* < 0.001

### Age-related changes in strength performance and strength-stability control in the dominant and non-dominant hands during the three manual precision tasks

**Fig. 2:**
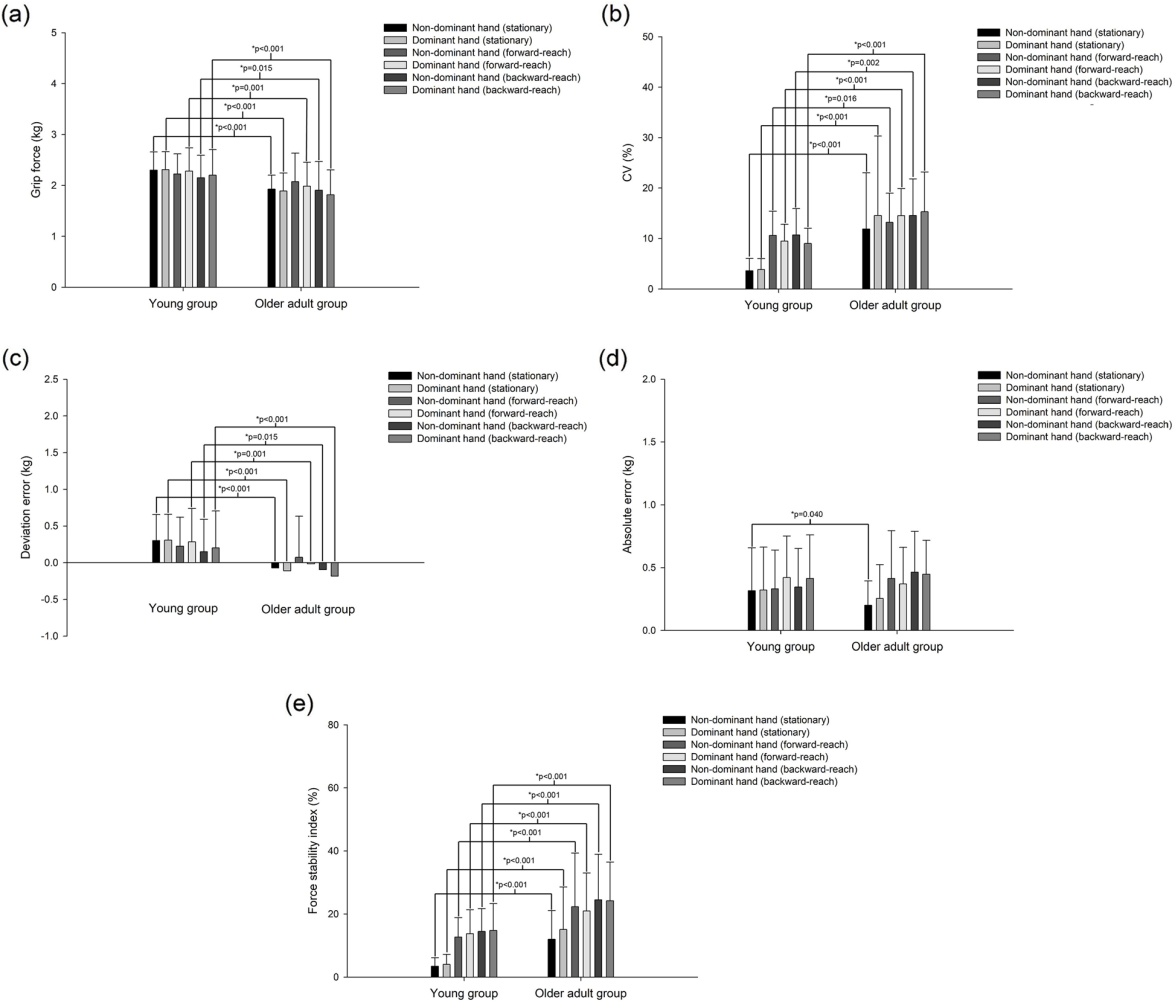
Age-related changes in grip force (**a**) and CV (**b**) for grip strength, and the deviation error (**c**), absolute error (**d**), and force-stability index (**e**) for strength-stability control in both hands during the three manual precision tasks. The error bars represent the standard deviation values

In the older adult group, except for the non-dominant hand under forward-reach conditions, grip force (Fig. 2a) and deviation error (Fig. 2c) values were significantly lower for the dominant and non-dominant hand during the three manual precision tasks than those in young adults (non-dominant hand under backward-reach conditions: *p* = 0.015, *p* = 0.015; other: all *p* < 0.001). Meanwhile, the CV (Fig. 2b) and force-stability index (Fig. 2e) values in the dominant and non-dominant hand were found to be significantly higher in the older adults during the three manual precision tasks than those in young adults (CV for non-dominant hand under forward-reach and backward-reach conditions: *p* = 0.016, *p* = 0.002; other: all *p* < 0.001).

### Grip-strength performance and stability control in the dominant and non-dominant hand during the three manual precision tasks for each group

Results showed that the different reaching movements significantly affected the CV for strength performance and the force-stability index values for strength-stability control for both hands in the young group (*p* < 0.001, Table 2). Post-hoc analysis also indicated that for both hands, the CV and force-stability index values under the forward- and backward-reach conditions were significantly higher than those in the stationary condition (*p* < 0.001, Table 2). Meanwhile, results also indicated that the different reaching movements significantly affected the absolute error and the force-stability index values for strength-stability control for both hands in older adult group (dominant hand: *p* = 0.002, *p* = 0.001; non-dominant hand: *p* < 0.001, *p* < 0.001, Table 3). Post-hoc analysis showed that the absolute error and force-stability index values under the backward-reach condition were significantly higher than those for the stationary condition in the dominant (*p* = 0.002, *p* = 0.001) and non-dominant (*p* < 0.001, *p* < 0.001) hand (Table 3). Furthermore, the absolute error and force-stability index values under the forward-reach condition were also significantly higher than those for the stationary condition in the non-dominant hand (*p* = 0.002, *p* = 0.001, Table 3).

**Table 2:** Comparison of grip-strength performance and stability control in the young group across the three manual precision tasks for the dominant and non-dominant hands

	Young group (***n*** = 50)					
	Dominant hand			Non-dominant hand		
	F value	Sig. (Two-tailed)	Scheffe post-hoc	F value	Sig. (Two-tailed)	Scheffe post-hoc
**Strength performance**						
Grip force (kg)	0.806	0.448		1.823	0.165	
CV (%)	57.833	< 0.001**	Condition 1 < Condition 2 (*p* < 0.001**) Condition 1 < Condition 3 (*p* < 0.001**)	44.356	< 0.001**	Condition 1 < Condition 2 (*p* < 0.001**) Condition 1 < Condition 3 (*p* < 0.001**)
**Strength-stability control**						
Deviation error (kg)	0.806	0.448		1.823	0.165	
Absolute error (kg)	1.295	0.277		0.099	0.905	
Force stability index (%)	37.789	< 0.001**	Condition 1 < Condition 2 (*p* < 0.001**) Condition 1 < Condition 3 (*p* < 0.001**)	53.262	< 0.001**	Condition 1 < Condition 2 (*p* < 0.001**) Condition 1 < Condition 3 (*p* < 0.001**)

*CV* coefficient of variation, *Diff* difference, *Sig* significance

*Significant difference *p* < 0.05

**Significant difference *p* < 0.001

**Table 3:** Comparison of grip-strength performance and stability control in the older adult group across the three tasks for the dominant and non-dominant hands

	Older adult group (***n*** = 54)					
	Dominant hand			Non-dominant hand		
	F value	Sig. (Two-tailed)	Scheffe post-hoc	F value	Sig. (Two-tailed)	Scheffe post-hoc
**Strength performance**						
Grip force (kg)	2.032	0.134		1.942	0.147	
CV (%)	0.072	0.931		1.230	0.295	
**Strength-stability control**						
Deviation error (kg)	2.032	0.134		1.942	0.147	
Absolute error (kg)	6.534	0.002*	Condition 1 < Condition 3 (*p* = 0.002*)	10.817	< 0.001**	Condition 1 < Condition 2 (*p* = 0.002*) Condition 1 < Condition 3 (*p* < 0.001**)
Force stability index (%)	7.104	0.001*	Condition 1 < Condition 3 (*p* = 0.001*)	12.286	< 0.001**	Condition 1 < Condition 2 (*p* = 0.001*) Condition 1 < Condition 3 (*p* < 0.001**)

*CV* coefficient of variation, *Diff* difference, *Sig* significance

*Significant difference *p* < 0.05

**Significant difference *p* < 0.001

## Discussion

In the present study, we chose the most common functional activity (combining the grip-strength generation and reaching movements) involving the upper extremities in daily living and analyzed the impact of aging and arm movement performance on grip-strength and grip-stability control. For both young and older adults, reaching movement performance resulted in reduced grip-force stability control (measured by CV values), decreased ability to sustain the target force (force-stability index values), and induced lower grip accuracy (absolute error) in both hands. Age-related deterioration in grip-force stability control (grip force and CV values) and the ability to sustain the target force (deviation error, absolute error, and force-stability index values) were found among older adults by comparing their scores during the three manual precision tasks with those of young adults. The results also showed that age and reaching-movement performance had an interaction effect on grip force and CV of muscle-strength performance and on the deviation error, absolute error, and force-stability index of strength-stability control. Based on these findings, this study indicated the older adults have inconsistent grip strength and instability control when performing arm-reaching movements.

### Quantitative measurement of grip-strength performance and stability control during manual precision tasks

Strain-gauge or load cell force transducers were used in previous studies to measure the relationship between grip-force generation and the load force of an object when asking healthy young participants to position their arms at specific postures or performing circular or lifting tasks [5–8, 27]. The findings provided valuable and quantitative information, revealing that the participants generated higher grip strength than the target force or the load force of the object (called the safety margin) to increase friction between the skin and the object and to prevent the target object from slipping [27]. These studies provided quantitative grip-force data in kg by calculating the deviation error values in grip performance during tasks. However, quantitative analysis of grip-strength and stability control regarding grip-strength performance of participants and of the variability of grip-stability control to the relevant target force of the object in both hands simultaneously is lacking in both young and older populations. In the present study, the CV values for grip-strength performance and deviation error and absolute error and force-stability index values for grip-stability control were calculated and applied to represent the participants’ grip-strength performance and grip-stability control in terms of the relevant target force. The CV value in the dominant hand was higher by 19.1% than that in the non-dominant hand during the backward-reach task for the young group (*p* = 0.017), and the absolute error value was also higher by 27.3% in the dominant hand than the non-dominant hand during the backward-reach condition (*p* = 0.049). Thus, the dominant hand generates higher grip strength than the non-dominant hand in relevant target force but is accompanied by unstable grip-strength generation for young, healthy adults. Furthermore, the older adult group showed a 25.6% higher force-stability index for the dominant hand than the non-dominant hand in the stationary condition (*p* = 0.035), representing poor grip force stability control in the dominant hand in the stationary condition. This poorer grip force stability control in the older population (by 15.3–17.7%) has been previously reported [10].

Additionally, compared with the grip-strength tests applied in previous studies [27], the grip-stability control tests in the present study were performed at 20% MVC target force; thus, the grip-force generated by both hands was much higher than the loading and lifting force of the dynamometer (dynamometer weight, 360 g). Consequently, young participants generated more grip strength, and the higher safety margins were also induced for both hands among three manual precision tasks by 0.15–0.31 kg (positive deviation error values). By contrast, the negative values for the deviation error to the target force for both hands were − 0.02 to − 0.18 kg during the three manual precision tasks. As such, the older adults instinctively decreased their grip strength to the relevant target force because they did not need to lift the dynamometer and generate higher grip strength to increase friction force to avoid slipping. If the older adults had generated a greater grip strength in terms of the load force of the object (i.e., creating a higher safety margin), it is likely that muscle fatigue would have been induced [28]. However, the age-related changes in grip strength and stability control among manual precision tasks could be induced and are a cause for concern in older adults.

### Influence of aging on grip-strength performance and stability control

The CV and force-stability index values indicated the age-induced deterioration in the quality of grip strength and stability control in both the dominant and non-dominant hands of older adults. Compared with the healthy young group, the older group showed higher CV and force-stability index values in both the dominant (5.1–10.7% and 7.2–11.1%, respectively) and non-dominant (2.6–8.3% and 8.5–10.1%, respectively) hands during the three manual precision tasks. These findings are consistent with those in previously reported studies reporting that age was the main factor affecting strength-stability control, and that older adults had higher variability in grip-strength performance and poorer stability control than did young adults during sustained grip effort, as indicated by the CV values from grip force data, representing the magnitude of force variability for each participant [22, 29]. This age-related change in grip-strength performance and strength stability control may have resulted from structural and functional degeneration in the central and peripheral nervous systems, deficits in perception feedback and receptors, and grip pattern dysfunction.

In terms of the physiology of brain function, several motor and sensory areas, including the contralateral primary motor cortex, primary sensory cortex, premotor cortex areas, bilateral prefrontal cortex, supplementary motor area, and cerebellum areas, are involved in the grip-strength generation of the hands [30]. In addition, as grip strength increases, the magnitude of neural excitation and the activation of regions in the ipsilateral supplementary motor area, globus pallidus internus, and the subthalamic nucleus also increase [30, 31]. However, with aging, atrophy of the motor cortical regions and corpus callosum [32] and reduction of grey matter and dendritic density [33] occur in the brain, which could result in motor and functional impairments in older adults [32]. Functional degeneration in the central nervous system may also occur with aging. For example, a previous study reported that neuron activation of the contralateral primary motor cortex, primary sensory cortex, posterior cingulate motor areas, and premotor cortex areas reduces with age, and that increasing neuron excitation in these areas improves grip-force levels in older adults [34]. In the present study, all participants were asked to execute grip-force stability control at 20% MVC during the three manual precision tasks; thus, when resistance was applied, the older adults could induce higher neuron activation and recruit more neural networks (ipsilateral primary motor cortex, putamen, subthalamic nuclei, substantia nigra, lateral globus pallidus, and contralateral cerebellum) than young adults [35]. Although degeneration of the central nervous system can cause poor strength performance and strength-stability control, other age-related changes of the peripheral nervous system may also affect grip strength and induce unstable grip-stability, reduce hand dexterity, and result in abnormal compensatory strategies and discoordination of the hands [12, 36]. Examples include the decline in nerve conduction and functions of the sensory system and the reduction in the number and sensitivity of somatosensory receptors in the skin, muscles, and joints [37]. In a previous study, a group of young adults was asked to hold an object and generate grip force at a target force level; these participants distributed their grip forces across all fingers to maintain stable grip-force strength and stability control [38]. However, with aging, the frequency, hand strength, and movement time of grip patterns significantly change [39], and antagonist muscle activation is also induced [40]; this phenomenon may result in discoordination of the grip-force generated by all fingers and reduce grip-stability control in older adults.

Grip-force generation is frequently associated with coordination movements involving both the upper extremities and simultaneous cognitive tasks [4]. This concurrent activity combining cognition and movement control was reported as postural (arm posture or movements) and suprapostural (stable grip strength generation and hold) tasks, which is one of the dual tasks described in previous studies [1–3]. Early studies have also revealed that the grip strength and moments are affected, and grip-force control in young people varies when assuming different postures or performing arm movements [2–5].

### Arm-reaching performance impacts grip-strength performance and stability control in young and older adults

The concurrent activity combining the cognitive attention involved in grip strength generation and arm reaching performance was reported as part of dual tasks in previous studies [1–3]. Many activities of daily living, such as walking and maintaining balance, are dual tasks [41] that involve the concurrent use of cognition, posture, and motor processes [42] and require the simultaneous application of several physiologic systems and cognition. In the present study, compared with that in the stationary condition, arm reaching performance resulted in inconsistent grip strength (higher CV values) in both the dominant (0.8–5.6%) and non-dominant hands (1.3–7.1%) of participants under forward- and backward-reach conditions. Poor grip-force stability control relative to the target force (higher force stability index values) was observed in both the dominant (5.9–10.8%) and non-dominant hands (9.2–12.5%) of both the young and older adult groups under forward- and backward-reach conditions, compared with that under the stationary condition. Several studies have also reported similar findings and revealed that grip force generation and control are unstable when positioning at specific postures or performing arm movement [5–8]. This phenomenon may be induced by shifting the attention resource from grip strength control to arm movements when performing grip-force stability control tests for the three manual precision tasks, and each manual precision task condition required different levels of effort. Additionally, attentional shifting can cause a delay in the onset time of movement, which increases movement time, induces compensative movements, and exacerbates the risk of accidents [41–44].

Previous studies have also indicated the impact of different tasks on grip-strength performance and reported varying responses when participants performed different dual tasks [43–47]. For example, previous studies have investigated motor or grip-force control among young and older people under the following conditions: performing a force-tracking task combined with an n-back test [45], executing a forward-reach task combined with posture change [46], reaching and grasping an object while performing a counting task [43], recovering balance to determine the reach-to-grip response [44], and gripping and lifting ability during a single-leg stance with the eyes closed [47]. The findings of these studies indicated that such different tasks resulted in poor grip strength, inaccurate performance, and high variability in repeated force-tracking [11, 21, 44–47]. Additionally, the compensatory mechanism for neuron network reorganization in older adults may be induced. For example, several additional areas of the bilateral hemispheres are recruited, and higher levels of neuron activation are generated [35, 48–50]. Previous studies also reported that the ability for neuron modulation in appropriate motor networks in the brain is reduced [34, 51] when older adults perform motor and cognition tasks. However, the potential impact of compensatory mechanisms on grip strength and stability control remains unknown and has rarely been discussed with regard to young and older populations. In the present study, results indicated changes in stability (CV value) and accuracy (force stability index value) in grip strength performance and stability control in both hands during forward- and backward-reaching movements among young adults and revealed insufficient grip strength and poor stability control relative to the target force in both hands among older adults. These findings may explain the real impact of compensatory mechanisms of both central neuron network and arm movements on grip strength and stability in young and older populations. Finally, age and arm-reaching conditions have an interaction effect in terms of CV in grip strength performance and force-stability index in stability control, which means that the older adults exhibited unstable grip strength during arm-reaching movements and this finding was statistically significant. Factors such as age, cognitive impairment, and frailty can also affect grip strength performance [44]. Therefore, we advise clinical therapists to develop appropriate health promotion exercises or rehabilitation strategies to achieve stable grip strength control during arm movements and prevent loss of grip on objects, for the avoidance of future accidents in this older age group.

### Study limitations and suggestions for future research

The findings of this study may help occupational and physical therapists develop appropriate rehabilitation programs or health-promotion exercises for improving grip strength performance and stability control in older people. Previous studies have indicated the correlation between age-related deterioration in grip strength and frailty and illness [14–17]. However, the present study did not recruit older people with disabilities or frailty and did not analyze the relationship between grip strength and stability control deficits and functional impairments of the hands and upper limbs. Future studies should recruit older people with disabilities or frailty, collect grip strength and stability control parameters, and conduct an upper extremity evaluation using clinical motor and functional assessment scales. It is important to analyze the relationship between these parameters and the clinical scale scores. Such investigations would generate further evidence-based data regarding the quality of grip strength among older adults with functional limitations and associated disorders of the upper extremities. Additionally, this study demonstrated unstable grip strength and stability control among arm movements in older adults compared with those in young adults by analyzing several grip-stability control indexes (CV, force stability index). Future studies should conduct direct measurements, collect continual grip-force data, and calculate the grip-stability control index to determine the quality of grip-strength generation during physical performance in frail and pre-frail elderly people, rather than only evaluating maximal grip force using traditional tests. These data should also be incorporated into comprehensive geriatric assessments during frailty screening processes. Furthermore, the purpose of this study was to evaluate the impact of aging and reaching movements on grip strength and stability control in both hands, and, to our knowledge, no study has reported that hand dominance is one of the factors that affect the grip stability control of young and older adults. Therefore, we did not perform full factorial analysis of Condition x Age x Hand in this study, and only separately analyzed the two-way Condition x Age interaction for both the dominant and non-dominant hand. However, aging, manual precision tasks, and hand dominance could have interaction effects on grip stability control, although future studies will be needed to assess these aspects. This may also help clinical therapists in developing appropriate rehabilitation programs or health-promotion exercises to improve grip-stability control during daily living activities in frail and pre-frail older people.

## Conclusions

This study indicated that aging and arm movement performance impacted the grip strength and stability control of both the dominant and non-dominant hands. The findings revealed that older adults developed reduced grip strength and stability control in both hands when they performed reaching movements involving the upper extremities.

## Data Availability

All data used and analyzed in this study are available from the corresponding author on reasonable request.

## Abbreviations

MVC: Maximal voluntary contraction
MMSE: Mini-mental status examination
CV: Coefficient of variation
CGA: Comprehensive geriatric assessment
SPSS: Statistical Package of the Social Sciences
ANCOVA: Analysis of covariance
